# Pyoverdine-Dependent Virulence of *Pseudomonas aeruginosa* Isolates From Cystic Fibrosis Patients

**DOI:** 10.3389/fmicb.2019.02048

**Published:** 2019-09-06

**Authors:** Donghoon Kang, Alexey V. Revtovich, Qingquan Chen, Kush N. Shah, Carolyn L. Cannon, Natalia V. Kirienko

**Affiliations:** ^1^Department of BioSciences, Rice University, Houston, TX, United States; ^2^Department of Microbial Pathogenesis and Immunology, Texas A&M University Health Science Center, College Station, TX, United States

**Keywords:** *Pseudomonas aeruginosa*, pyoverdine, cystic fibrosis, *Caenorhabditis elegans*, murine infection, antivirulence, antimicrobial resistance

## Abstract

The development of therapies that modulate or prevent pathogen virulence may be a key strategy for circumventing antimicrobial resistance. Toward that end, we examined the production of pyoverdine, a key virulence determinant, in ∼70 *Pseudomonas aeruginosa* isolates from pediatric cystic fibrosis patients. Pyoverdine production was heterogeneous and showed a clear correlation with pathogenicity in *Caenorhabditis elegans* and an acute murine pneumonia model. Examination showed pyoverdine accumulation in host tissues, including extrapharyngeal tissues of *C. elegans* and lung tissues of mice, where accumulation correlated with host death. Many of the isolates tested were resistant to multiple antimicrobials, so we assayed the ability of pyoverdine inhibitors to mitigate virulence and rescue pyoverdine-mediated host pathology. Representatives from three different classes of pyoverdine inhibitors (gallium, fluoropyrimidines, and LK11) significantly improved survival. Our findings highlight the utility of targeting virulence factors in general, and pyoverdine in particular, as a promising method to control bacterial pathogenesis as the utility of antimicrobials continues to diminish.

## Introduction

*Pseudomonas aeruginosa* is a Gram-negative opportunistic pathogen that causes life-threatening infections in immunocompromised patients, particularly those with cystic fibrosis (CF). CF is a monogenic disorder caused by mutations in the Cystic Fibrosis Transmembrane conductance Regulator (CFTR). Although the precise mechanism remains unclear, dysfunctional CFTR results in abnormally thick and viscous mucus lining the respiratory epithelium, probably due to improper hydration of the mucosal layer (Lyczak et al., 2002). Increased mucus viscosity compromises the effective removal of bacteria and other trapped particles, allowing organisms to colonize and persist. Once established, *P. aeruginosa* activates genetic pathways that support a transition from an acute to a chronic infection profile, which is associated with increased resistance to most host defense strategies (Furukawa et al., 2006; Winstanley et al., 2016). By the age of 30, over 70% of CF patients battle chronic pseudomonal infections, which are extremely difficult to eradicate (Sorde et al., 2011). These infections typically trigger the decline of respiratory function in CF patients, especially children, and significantly contribute to the high mortality rates observed in this patient population (Kosorok et al., 2001; Emerson et al., 2002).

Even in non-CF populations, *P. aeruginosa* infections are intrinsically difficult to treat. For example, *P. aeruginosa* is a leading cause of infectious death in immunocompromised leukemia patients receiving chemotherapy (O’Connor et al., 2014). Due to the spread of multidrug resistance (MDR), even environmental strains of *P. aeruginosa* are often resistant to several antimicrobials, and clinical isolates are frequently extensively resistant. Even pandrug-resistant strains have been observed for over 15 years, and mortality rates for patients infected with these strains approach 75% (Wang et al., 2006).

The rise in MDR pathogen infection rates has galvanized efforts to identify new ways to prevent or treat bacterial infections. One promising new therapeutic approach is to target bacterial virulence factors, with the goal of limiting or preventing pathology rather than outright killing the bacteria. In many cases, the bacterial products that cause host damage are dispensable for colonization or even growth, and their absence leads pathogens to develop a more commensal profile (Johnson and Abramovitch, 2017). Attenuation of virulence may also help the host’s natural defenses to mitigate pathogenesis and clear the infection, particularly if the virulence factor(s) targeted is/are required for more than one mechanism of pathogenesis.

In most biologically relevant contexts, iron is present at concentrations far too low for biological needs. Iron restriction is even a key method used by most multicellular organisms to limit infection (Hood and Skaar, 2012). Consequently, most microorganisms have developed complex methods to obtain the iron they require, including the production of water-soluble, iron-chelating molecules known as siderophores. Pyoverdine, the major siderophore produced by *P. aeruginosa*, is a good example. In addition to solubilizing iron from inorganic sources, pyoverdine can remove this metal from host iron-sequestering factors like transferrin (Meyer et al., 1996) and from ferroproteins resident in host mitochondria, disrupting their biology and triggering mitochondrial surveillance pathways (Kirienko et al., 2013, 2015; Kang et al., 2018). Once, it has acquired iron, pyoverdine functions as a signaling molecule to regulate the production of several secreted toxins (Ochsner et al., 1996; Lamont et al., 2002). When ferripyoverdine binds to its receptor, FpvA, in the outer membrane of the bacterium, an alternative sigma factor, PvdS, is released from sequestration on the inner membrane (Beare et al., 2003). PvdS then activates production of the translational inhibitor Exotoxin A, the protease PrpL, and genes responsible for pyoverdine biosynthesis. A combination of these functions makes pyoverdine production necessary for full *P. aeruginosa* virulence against both mammalian and invertebrate hosts (Meyer et al., 1996; Takase et al., 2000; Kirienko et al., 2013; Minandri et al., 2016). Compromising pyoverdine, whether by blocking its production with fluoropyrimidines (Imperi et al., 2013; Kirienko et al., 2016) or its function using gallium (Ga^3+^) or pyoverdine inhibitors (Kaneko et al., 2007; Kirienko et al., 2019), is sufficient to mitigate *P. aeruginosa* pathogenesis, validating pyoverdine as a therapeutic target.

In this study, we recapitulated findings from previous murine infection studies using a highly virulent strain of *P. aeruginosa* (PA14) and showed that chemical or genetic disruption of pyoverdine production reduces virulence. Furthermore, we surveyed multidrug-resistant strains of *P. aeruginosa* isolated from CF patients and demonstrated that there is a strong, positive correlation between pyoverdine production and virulence against *Caenorhabditis elegans*. We were able to recapitulate these results in a murine pneumonia model by directly measuring *in vivo* pyoverdine production. Finally, we demonstrated that previously characterized pyoverdine inhibitors can be used to effectively mitigate pathogenesis of multidrug-resistant and otherwise highly virulent *P. aeruginosa* isolates from CF patients.

## Materials and Methods

### Bacteria and *C. elegans* Strains

*Pseudomonas aeruginosa* PA14 (Mathee, 2018; Schroth et al., 2018) and PA14*pvdF* (Liberati et al., 2006) were provided by Dr. Fredrick Ausubel. All *P. aeruginosa* isolates were obtained from the sputa of pediatric CF patients at St. Louis Children’s Hospital and routinely banked by the clinical microbiology laboratory. The de-identified isolates were provided to Dr. Cannon. Isolates’ species identity was confirmed and antimicrobial susceptibility was determined by the Johns Hopkins Hospital microbiology laboratory. Antimicrobial susceptibility was determined according to CLSI standards through the use of the BD Phoenix^TM^ automated identification and susceptibility testing system. The antimicrobial susceptibilities of isolates that failed to grow in the Phoenix system were determined using conventional disk susceptibility testing. For *C. elegans* assays, the temperature-sterile strain SS104 [*glp-4(bn2)*] was used (Beanan and Strome, 1992).

### Acute Murine-*P. aeruginosa* Lung Infection Model

Male C57BL/6J mice (The Jackson Laboratory, Bar Harbor, ME, United States) aged 6 to 8 weeks were used for all acute lung infection studies (Cannon et al., 2009), which were approved by the Texas A&M University Institutional Animal Care and Use Committee (IACUC). Mice were weighed and randomly assigned into groups and were housed in a barrier facility under pathogen-free conditions until bacterial inoculation. When necessary, animals were euthanized with an overdose of ketamine:xylazine followed by cardiac puncture for exsanguination, a method approved by the IACUC (TAMU) and consistent with the recommendations of the Panel on Euthanasia of the American Veterinary Medical Association.

*Pseudomonas aeruginosa* isolates were grown to an OD_650_ of 0.4 in LB (LB), pelleted, washed with phosphate buffered saline (PBS), and resuspended to an OD_650_ of 0.4 in LB (corresponding 3.3 × 10^6^ CFU/mL, as determined by serial dilution and plating). Initial experiments determined that the lowest LD_100_ dose for the wild-type *P. aeruginosa* strain PA14 is between 2.5 and 3.5 × 10^5^ CFU. This inoculum dose was subsequently used for all of the *P. aeruginosa* isolates in all of the experiments.

Following anesthesia via intraperitoneal injection of ketamine (60 mg/kg) and xylazine (8 mg/kg) cocktail, mice were intranasally inoculated with 75 μL of bacteria in LB broth at an LD_100_ of ∼2.5 × 10^5^ CFU per mouse. To test the efficacy of 5-fluorocytosine (5-FC) as a pyoverdine inhibitor, 2 h post-infection with PA14, and every 12 h subsequently for a maximum of six doses, mice were intraperitoneally injected with either 2.5 μM of 5-FC (50 μL of a 50 mM stock solution in water with 5% DMSO) or with saline in water with 5% DMSO. Mice were not anesthetized for injection, but rather briefly cradled supine. Infected mice were weighed and assigned a clinical infection score (Shah et al., 2018) every 24 h post-infection. A clinical score is a semi-quantitative metric that evaluates for signs of infection, and ranges from asymptomatic (score 0) to moribund (score 6) based on the resting posture (0–2), coat (0–1), and activity level (0–3) of the infected mice. Finally, as soon after death as was feasible, or after euthanasia of the survivors, lungs were harvested and homogenized to establish pyoverdine content in the tissue. The experiment was performed twice and the data were pooled.

### *C. elegans* Liquid Killing

Liquid Killing assay was performed as previously described (Anderson et al., 2018). In brief, *C. elegans* were exposed to overnight cultures of *P. aeruginosa* diluted to OD_600_ 0.03 in Liquid Kill media [a 1:4 mix of SK media (0.3% w/v NaCl, 0.35% w/v Bacto-Peptone in water) and S-Basal (100 mM NaCl, 50 mM potassium phosphate, pH 6.0)] in 384-well plates. After exposure, *C. elegans* were stained with Sytox Orange (Fisher Scientific). Fluorescent images were acquired using a BioTek Cytation5 multi-mode plate reader (BioTek) and analyzed via Cell Profiler^1^.

### *P. aeruginosa* Biofilm Assays

Overnight cultures of *P. aeruginosa* grown in LB were first diluted 20-fold into 1 mL of M9 media [1% 5X M9 salts (Difco), 1.3% Casamino Acids (Difco), 1 mM CaCl_2_, 1 mM MgSO_4_] in 12-well plates. Bacteria were statically grown at 30 °C for 16 h. Biofilms were stained with 0.1% (w/v) crystal violet in 20% (v/v) ethanol/water for 30 min. Excess stain was washed with PBS (Gibco). Remaining stain in biofilms was solubilized in 30% acetic acid and was quantified by absorbance at 550 nm.

To visualize pyoverdine production in biofilm matrix cells, bacteria were grown under conditions as above. The multiwell plate was washed with PBS to remove planktonic bacteria and pyoverdine in the supernatant. Biofilm matrices were imaged using a pyoverdine-specific filter (Kang and Kirienko, 2017) in a BioTek Cytation5 multi-mode plate reader.

### Pyoverdine-Rich Bacterial Filtrates

Overnight cultures of *P. aeruginosa* grown in LB medium were inoculated into 30 mL of modified M9 media [1% 5X M9 salts (Difco), 1.3% Casamino Acids (low sodium chloride and iron) (Difco), 1 mM CaCl_2_, and 1 mM MgSO_4_] containing low iron. Bacteria were grown in 250 mL flasks that were shaken at 225 rpm at 37 °C for 12 h. The resulting culture was centrifuged to remove bacteria and supernatants were filtered through a 0.22 μm pore membrane at least twice. Gentamicin was added to a final concentration of 100 μg/mL to maintain sterility.

### Confocal Microscopy

Pyoverdine was visualized within *C. elegans* as previously described (Kang et al., 2018). Briefly, worms were exposed to pyoverdine-rich bacterial filtrates for 24 h. Afterward, worms were extensively washed in S Basal buffer, paralyzed with 25 mM levamisole hydrochloride, and imaged under a Zeiss LSM 710 laser-scanning confocal microscope using a 405 nm argon laser (Kang et al., 2018).

### Inductively Coupled Plasma Mass Spectrometry (ICP-MS)

Inductively coupled plasma mass spectrometry (ICP-MS) of *C. elegans* treated with *P. aeruginosa* were performed as previously described (Kang et al., 2018). Briefly, *C. elegans* were exposed to *P. aeruginosa* isolates PA2-61, PA2-72, PA2-88, or PA3-22 in SK media for 65 h at 25 °C. Worms were collected and organic material was removed via overnight digestion using 3 mL of 69% trace-metals grade nitric acid. Digestions (including worm-free controls) were performed in 20 mL glass scintillation vials (Wheaton) overnight on a heat block set to 95 °C. Digestions were performed to dryness at least four times, which results in the complete degeneration of organic molecules that can foul the ICP-MS. The remaining inorganic material was then reconstituted in 2% nitric acid (v/v), 2% ethanol (v/v) containing indium and yttrium as internal standards. Iron concentration was quantified on a Perkin Elmer Nexion 300X ICP-MS system and compared to a seven point calibration curve that had a correlation coefficient > 0.999.

### Siderotyping by High Performance Liquid Chromatography (HPLC)

Pyoverdine-rich bacterial filtrates of *P. aeruginosa* strains with defined pyoverdine structures (Type I – PAO1, PA14; Type II – ATCC27853; Type III – ATCC25010, ATCC33360) and uncharacterized CF isolates were produced. Filtrates were passed through a 3 kD centrifugal filter (Fisher Scientific) to remove large molecules. Bacterial filtrates were analyzed on a 1220 Infinity LC system (Agilent) with a peptide XB-C18 analytical column (Aeris). HPLC was performed on an acetonitrile (0.1% trifluoroacetic acid) gradient from 5 to 65% (in water) in 30 min.

### Statistical Analysis

For murine infection experiments, analyses were performed using Prism 7 (GraphPad Software, Inc.). *In vivo* survival curves were compared using a Log-rank Mantel–Cox test, differences in weight change and clinical scores were analyzed using Kruskal–Wallis one-way ANOVA and Dunn’s multiple comparison test. Data were deemed to be significantly different for *p* ≤ 0.05.

## Results

### Pyoverdine Production Is Necessary for Full *P. aeruginosa* Virulence in a Murine Pneumonia Model

**FIGURE 1:**
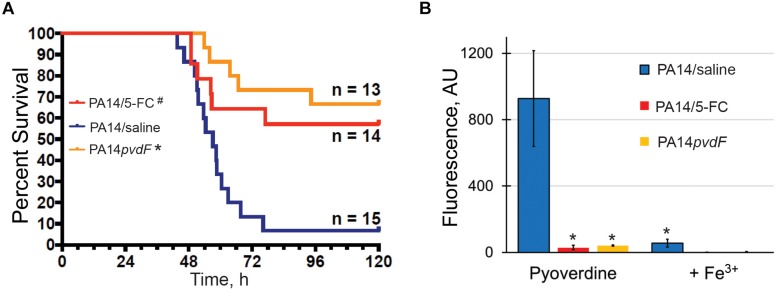
Pyoverdine production is necessary for *P. aeruginosa* virulence in a murine acute pneumonia model. **(A)** Survival advantage over sham (PA14: saline) in mice treated with 5-fluorocytosine (5-FC) or those inoculated with PA14*pvdF*. All drug and saline doses contained 5% DMSO. Data were pooled from two replicates. *p-*Values were calculated using Kruskal–Wallis one-way ANOVA and Dunn’s multiple comparison test. ^#^*p* < 0.01, ^∗^*p* < 0.0001 compared to saline control. **(B)** Pyoverdine content in mouse lung homogenates. Pyoverdine fluorescence was measured after treatment with 8-hydroxyquinoline to remove iron from ferripyoverdine. Error bars represent sample standard deviation. *p*-Values were calculated using Student’s *t*-test. ^∗^*p* < 0.01.

To investigate the significance of pyoverdine production during *P. aeruginosa* pathogenesis, we infected mice with wild-type *P. aeruginosa* PA14 or an isogenic pyoverdine biosynthetic mutant (*P. aeruginosa* PA14*pvdF*) via intranasal inoculation and monitored host health and survival. Consistent with previous studies (Meyer et al., 1996; Takase et al., 2000; Minandri et al., 2016), mice infected with the mutant strain deficient for pyoverdine production demonstrated improved survival, less weight loss, and lower clinical scores than mice infected with wild-type *P. aeruginosa* (Figure 1A and Supplementary Figures S1A,B). Importantly, our results were obtained with *P. aeruginosa* PA14, a more virulent strain than PAO1, which was used in previous reports. We verified pyoverdine production *in vivo* by homogenizing lung tissue and measuring pyoverdine content in the homogenate. Pyoverdine’s characteristic fluorescence (Em. 405 nm; Ex. 460 nm) was seen in the lung tissue of mice infected with wild-type PA14, but not in those infected with PA14*pvdF*. This fluorescence was quenched by the addition of ferric iron (Figure 1B), corroborating that it was pyoverdine-derived. Mice treated with 5-fluorocytosine, an inhibitor of pyoverdine biosynthesis (Imperi et al., 2013; Kirienko et al., 2016), did not accumulate pyoverdine and died at a substantially lower rate during infection (Figures 1A,B), reiterating the importance of this virulence factor for pathogenesis.

### Pyoverdine Production Correlates With Virulence in Isolates From Cystic Fibrosis Patients

**FIGURE 2:**
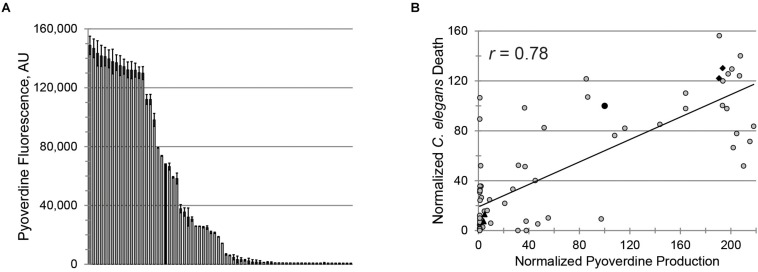
Pyoverdine production correlates with virulence against *Caenorhabditis elegans*. **(A)** Pyoverdine production in 69 *P. aeruginosa* isolates from CF patients (black bar: PA14). Error bars represent SEM from two biological replicates. **(B)** Correlation between pyoverdine production (from bacteria statically grown in M9 media) and *C. elegans* death after *P. aeruginosa* exposure (in Liquid Killing conditions) – see section “Materials and Methods” for details. Data were normalized to PA14. Black diamonds and triangles represent the high- and low-virulence isolates selected for further study. Each point represents the average of at least two biological replicates.

To evaluate the clinical utility of targeting pyoverdine to mitigate pathogenesis, we investigated the relationship between pyoverdine production and virulence in 69 isolates of *P. aeruginosa* obtained from pediatric patients with CF (Supplementary Table S1). Consistent with other reports (De Vos et al., 2001; Ernst et al., 2003; Smith et al., 2006; Martin et al., 2011; Marvig et al., 2014; Nguyen et al., 2014; Andersen et al., 2015), pyoverdine production in these isolates was heterogeneous, with ∼30% producing more pyoverdine than PA14 and ∼30% exhibiting virtually no pyoverdine production (Figure 2A).

*Pseudomonas aeruginosa* virulence was measured in a *C. elegans* Liquid Killing model where pyoverdine has been identified as a key factor (Kirienko et al., 2013). As expected, we observed a strong, statistically significant, positive correlation between pyoverdine production and virulence (Figure 2B). Interestingly, the correlation between biofilm production and virulence or between production of biofilm and pyoverdine was weaker (Supplementary Figures S2A,B). The absence of strong correlation between biofilm and pyoverdine was somewhat unexpected considering that previous studies by our lab and others have demonstrated that biofilm formation is necessary for pyoverdine production (Visaggio et al., 2015; Kang and Kirienko, 2017, 2018). One possible explanation for this observation was that some isolates exhibit unusual aggregates that promote pyoverdine production, even in the absence of *bona fide* biofilm (Supplementary Figures S2C–E) (Kang et al., 2017). In strains that form more typical biofilms, we were able to visualize cell aggregates attached to the well (Supplementary Figure S2C). In isolates that produced atypical biofilms, aggregates were not attached to the surfaces of the growth vessel (Supplementary Figure S2D). However, these clusters were still associated with high levels of pyoverdine, as shown by the presence of characteristic pyoverdine fluorescence (Supplementary Figure S2E). Based on virulence and pyoverdine production, two isolates were chosen to represent highly virulent, high pyoverdine production (PA2-61, PA2-72) and avirulent, low pyoverdine production states (PA2-88, PA3-22), as indicated (Figure 2B).

### Pyoverdine From High-Virulence Isolates Can Translocate Into *C. elegans*

**FIGURE 3:**
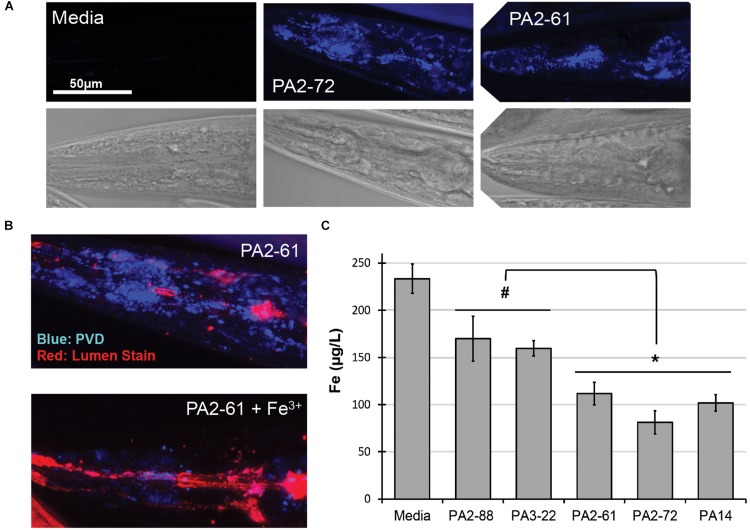
Pyoverdine from virulent isolates translocates into *C. elegans* and disrupts host iron homeostasis. **(A)** Confocal laser-scanning micrographs of *C. elegans* pharynx in worms treated with media or pyoverdine-rich bacterial filtrates from PA2-61 and PA2-72. Pyoverdine fluorescence is shown in blue. **(B)** Pyoverdine accumulation in extralumenal pharyngeal tissue after exposure to filtrate from PA2-61 or the same filtrate pre-saturated with ferric iron. Blue indicates fluorescence from iron-free pyoverdine, red staining shows the pharyngeal lumen. **(C)** *C. elegans* iron content after exposure to highly virulent (PA2-61, PA2-72, PA14) and avirulent (PA2-88, PA3-22) isolates or media in the absence of pathogen. Host iron content was measured via inductively coupled plasma mass spectrometry (ICP-MS) (see Supplementary Materials and Methods). Error bars represent SEM between three biological replicates. *p*-Values were calculated using Student’s *t*-test. ^#^*p* < 0.05, ^∗^*p* < 0.01.

We previously reported that pyoverdine from *P. aeruginosa* PA14 can translocate into *C. elegans*, where it chelates intracellular iron (Kang et al., 2018). To test whether pyoverdines from other high-virulence isolates share this ability, worms were exposed to pyoverdine-rich, bacteria-free, spent growth media (hereafter referred to as filtrates) from PA2-61 and PA2-72. Fluorescence characteristic of pyoverdine was found in extralumenal tissues surrounding the pharynges of *C. elegans* (Figure 3A) as was previously seen for pyoverdine from PA14 (Kang et al., 2018). This fluorescence was attenuated when the filtrate was pre-treated with ferric iron, which quenches pyoverdine (Figure 3B). Furthermore, consistent with pyoverdine being a strong iron chelator that can translocate in and out of *C. elegans*, worms treated with high-virulence isolates PA2-61 or PA2-72 exhibited greater iron loss during Liquid Killing than those treated with pyoverdine-deficient isolates PA2-88 or PA3-22, as measured by ICP-MS (Figure 3C). It is important to note, however, that even *C. elegans* treated with pyoverdine-deficient isolates exhibited a decrease in iron content compared to the uninfected controls (Figure 3C).

### Pyoverdine Production Predicts Virulence in a Murine Pneumonia Model

**FIGURE 4:**
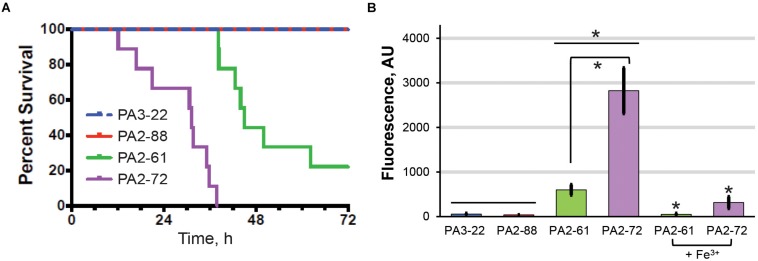
*In vivo* pyoverdine production correlates with virulence in murine hosts. **(A)** Mice survival after intranasal inoculation with strains that were highly virulent (PA2-61, PA2-72) or avirulent (PA2-88, PA 3-22) against *C. elegans*. **(B)** Pyoverdine content in mouse lung homogenates. Pyoverdine fluorescence was measured after 8-hydroxyquinoline treatment to remove iron from ferripyoverdine. Error bars represent sample standard deviation. Data were pooled from two replicates. *p-*values in **(A)** were calculated using Kruskal–Wallis one-way ANOVA and Dunn’s multiple comparison test. *p* < 0.01 for PA2-61 vs. PA2-88 or PA3-22, and PA2-72 vs. PA2-88 or PA3-22; *p* < 0.05 for PA2-61 vs. PA2-72. *p*-Values in **(B)** were calculated using Student’s *t*-test. ^∗^*p* < 0.01.

The high virulence, high-pyoverdine and avirulent, low-pyoverdine CF isolates were used to analyze the relationship between pyoverdine production and virulence in a murine pneumonia model. Consistent with results from the *C. elegans* assay, mice infected with PA2-61 or PA2-72 showed dramatically lower survival, greater weight loss, and higher clinical scores than those infected with PA2-88 or PA3-22 (Figure 4A and Supplementary Figures S3A,B). Importantly, host survival correlated with *in vivo* pyoverdine production. While the lungs of mice infected with the two virulent strains show significant accumulation of pyoverdine, there was no pyoverdine detectable in the lung tissue of mice infected with the two avirulent strains (Figure 4B). These data demonstrated two important findings. First, based on the isolates tested, pyoverdine production *in vitro* and *in vivo* correlate well, allowing *in vitro* measurements to serve as a simple method of predicting virulence. Second, the level of pyoverdine production and accumulation in the lungs correlated with the severity of disease, including survival rate and clinical score (Figures 4A,B). These results support the correlation between pyoverdine and virulence observed in the *C. elegans* pathosystem and reinforce the potential clinical utility of targeting pyoverdine during infection (as shown in Figure 1).

### Pyoverdine Inhibitors More Effectively Protect the Host From MDR *P. aeruginosa* Isolates Than Conventional Antimicrobials

**FIGURE 5:**
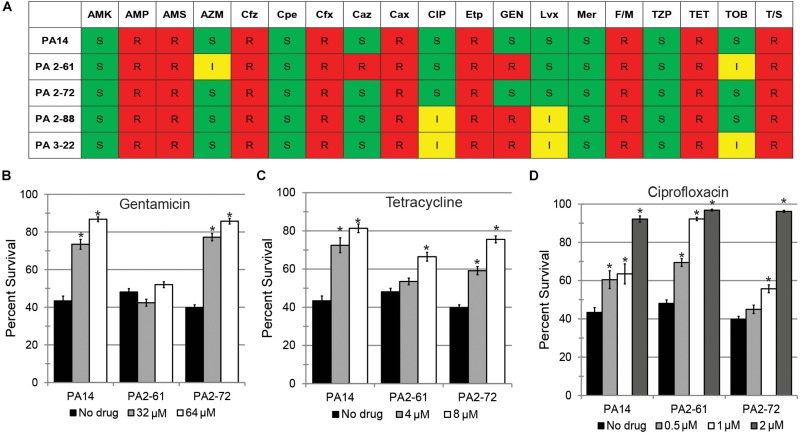
Conventional antibiotics display reduced efficacy against MDR isolates in a *C. elegans* model. **(A)** Susceptibility of representative virulent (PA14, PA2-61, PA2-72) and avirulent isolates (PA2-88, PA3-22) to various antibiotics, including AMK, amikacin; AMP, ampicillin; AMS, ampicillin-sulbactam; AZM, aztreonam; Cfz, cefazolin; Cpe, cefepime; Cfx, cefoxitin; Caz, ceftazidime; Cax, ceftriaxone; **CIP, ciprofloxacin**; Etp, ertapenem; **GEN, Gentamicin**; Imp, imipenem; Lvx, levofloxacin; Mer, meropenem; F/M, nitrofurantoin; TZP, piperacillin-tazobactam; **TET, tetracycline**; TOB, tobramycin; T/S, trimethoprim-sulfamethoxazole. Based on CLSI MIC cutpoints, S, I, and R indicate sensitivity, intermediate resistance, and complete resistance to the tested antimicrobial, respectively. *P. aeruginosa* is considered sensitive to CIP if the MIC is 1 μg/mL (∼2.6 μM) or less and sensitive to GEN if the MIC is 4 μg/mL (∼8.4 μM) or less. Most strains of *P. aeruginosa* are considered resistant to TET, but *Staphylococcus aureus* is considered sensitive if the MIC is 4 μg/mL (∼8.4 μM) or less. *C. elegans* survival after exposure to *P. aeruginosa* PA14, PA2-61, or PA2-72 in the presence of **(B)** gentamicin, **(C)** tetracycline, or **(D)** ciprofloxacin. Error bars represent SEM between three biological replicates. *p*-values were calculated using Student’s *t*-test. ^∗^*p* < 0.01.

Treatment of *P. aeruginosa* infections with antimicrobials is frequently inefficient because of the expression of multidrug efflux pumps and the formation of biofilm that limits physical access (Anderson and O’Toole, 2008). Each of the CF isolates were tested for their antimicrobial sensitivity. Most of the isolates were resistant to several antibiotics (Figure 5A and Supplementary Table S1). Inappropriate antimicrobial dosages can promote the emergence of resistant strains or even increase the production of virulence factors (Linares et al., 2006; Olson et al., 2017). Therefore, we tested whether three conventional antimicrobials (gentamicin, tetracycline, and ciprofloxacin) could effectively rescue *C. elegans*. Among the three virulent strains (PA14, PA2-61, and PA2-72), only PA2-61 was clinically resistant to gentamicin. This was recapitulated in our assay conditions, where even exceedingly high concentrations of the antibiotic (64 μM) had no effect on PA2-61 growth (Supplementary Figure S4A). Consistently, gentamicin treatment rescued *C. elegans* exposed to PA14 and PA2-72, but not PA2-61 (Figure 5B). Although all three isolates were clinically resistant to tetracycline and ciprofloxacin, pathogenesis was sometimes attenuated by the presence of the drugs (tetracycline for PA2-61 and ciprofloxacin for PA2-72, Figures 5C,D). As expected, antimicrobial concentrations that limited pathogenesis also inhibited bacterial growth, while no overt signs of bactericidal or bacteriostatic effects were seen at concentrations that did not rescue *C. elegans* (Supplementary Figure S4).

**FIGURE 6:**
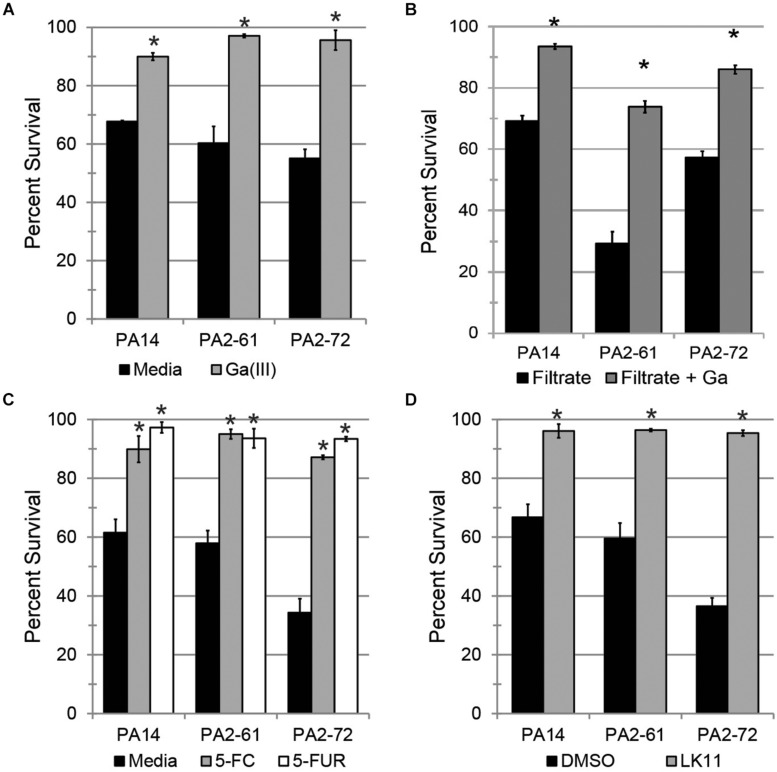
Pyoverdine inhibitors effectively mitigate *P. aeruginosa* pathogenesis. **(A)** *C. elegans* survival after exposure to *P. aeruginosa* PA14, PA2-61, or PA2-72 in the presence of 100 μM gallium. **(B)** *C. elegans* survival after exposure to pyoverdine-rich bacterial filtrates pre-saturated with gallium. **(C,D)** *C. elegans* survival after exposure to *P. aeruginosa* in the presence of **(C)** 50 μM 5-fluorocytosine (5-FC) and 10 μM 5-fluorouridine (5-FUR), or **(D)** 100 μM LK11. Error bars in **(A,C,D)** represent SEM between three biological replicates. Data presented in **(B)** are one representative result from three biological replicates; error bars represent SEM from 16 technical replicates. *p*-values were calculated using Student’s *t*-test. ^∗^*p* < 0.01.

Since PA2-61 and PA2-72 caused severe pathology in mice and displayed considerable resistance to antimicrobials, three pyoverdine-inhibiting antivirulents were tested for the ability to ameliorate their pathogenesis in the Liquid Killing model. Gallium (Ga^3+^), a redox-inactive trivalent cation, has an ionic radius very close to that of iron, allowing Ga^3+^ to be coordinated by iron-binding molecules, such as ferroproteins or pyoverdine. 100 μM Ga^3+^ effectively limited pathogenesis by all three strains tested (Figure 6A). However, gallium is known to have pleiotropic effects as it can be inappropriately incorporated in place of iron in many biological contexts, causing a variety of non-specific effects on the pathogen, including inhibiting growth. To eliminate this potential confound, pyoverdine-rich bacteria-free filtrates were prepared. These were split, and one-half was pre-saturated with gallium. The addition of gallium in this fashion rescued *C. elegans* from siderophore toxicity (Figure 6B). This is consistent with previous reports of the ability of gallium to disrupt pyoverdine function (Kaneko et al., 2007) and indicates that its effect is not solely on the bacterium. These results were also consistent with our previous data that pyoverdine is a critical virulence factor under these conditions (Kirienko et al., 2013; Kang et al., 2018).

Fluoropyrimidines (including 5-fluorouracil, 5-fluorocytosine, and 5-fluorouridine) have been shown to inhibit pyoverdine biosynthesis, improving survival of both *C. elegans* and mice (Figure 1A) (Imperi et al., 2013; Kirienko et al., 2016). Consistent with this, 50 μM of 5-fluorocytosine or 10 μM 5-fluorouridine were sufficient to rescue *C. elegans* (Figure 6C).

The newest approach, reported earlier this year (Kirienko et al., 2019), is based on inhibiting pyoverdine’s function, likely by preventing its interaction with iron. Previously, we have reported several molecules that have the ability to rescue *C. elegans* from *P. aeruginosa* strain PA14 (Kirienko et al., 2019). One such compound, LK11, was tested against the MDR clinical strains PA2-61 and PA2-72 (Figure 6D). LK11 was able to attenuate the virulence of both strains despite the fact that they produce different types of pyoverdine (PA2-61 produces type I while PA2-72 makes type II) (Kang et al., 2018; Supplementary Figure S5). These types of pyoverdines have distinct structures and chemical compositions (Cézard et al., 2015), demonstrating the broad clinical applicability of this molecule against a variety of *P. aeruginosa* strains. It is important to note that fluoropyrimidines and LK11 did not exhibit antimicrobial activity at the concentrations tested (Kirienko et al., 2016, 2019). Furthermore, their ability to rescue *C. elegans* from *P. aeruginosa* has been previously shown to depend on their mitigation of pyoverdine; the virulence of a pyoverdine biosynthetic mutant PA14*pvdF* was unchanged (Kirienko et al., 2016, 2019).

## Discussion

Using ∼70 *P. aeruginosa* isolates from pediatric CF patients, we probed the association of pyoverdine production and virulence. We saw a positive correlation between pyoverdine production and virulence against *C. elegans* and in a murine pneumonia model. Treatment that prevented pyoverdine biosynthesis (fluoropyrimidines) or pyoverdine function (gallium, LK11) ameliorated pyverdine-dependent pathology and rescued hosts.

Interestingly, infection with *P. aeruginosa* caused pyoverdine to accumulate in the lungs, and this pyoverdine concentration correlated with host death. Admittedly, these observations were made with a limited number of isolates and animals, but it does suggest that further investigation into the consequences of pyoverdine in the respiratory tract are warranted, particularly in the context of CF. Unfortunately, the role and importance of pyoverdine in CF patients remains unclear. Some studies, including the largest longitudinal, multicenter survey, demonstrate that CF patients harbor pyoverdine-producing strains and retain pyoverdine in their sputum (Haas et al., 1991; De Vos et al., 2001; Martin et al., 2011; Marvig et al., 2014; Mayer-Hamblett et al., 2014). Other studies show that at least some chronically infected patients harbor strains with mutated pyoverdine biosynthesis genes and reduced pyoverdine production (Ernst et al., 2003; Smith et al., 2006; Nguyen et al., 2014; Andersen et al., 2015), although a longitudinal study using *P. aeruginosa* PA14 (Marvig et al., 2014) did not find this result. Amongst the isolates we tested, the amount of pyoverdine produced varied remarkably, but about 2/3 of the strains had appreciable levels of pyoverdine.

Given the general requirement for pathogens to acquire iron from their hosts, it seems peculiar that bacteria would choose to abandon an effective route for its acquisition. There are several possible explanations for this phenomenon. First, pyoverdine production is not a prerequisite for pyoverdine utilization; most available evidence suggests that even strains that have lost the ability to produce pyoverdine (“cheaters”) retain one or more ferripyoverdine receptors (De Vos et al., 2001; Martin et al., 2011; Marvig et al., 2014; Andersen et al., 2015). Since pyoverdine production is energetically expensive, cheating confers a fitness benefit (Visca et al., 2007; Bruce et al., 2017; O’Brien et al., 2017; Weigert and Kummerli, 2017).

**FIGURE 7:**
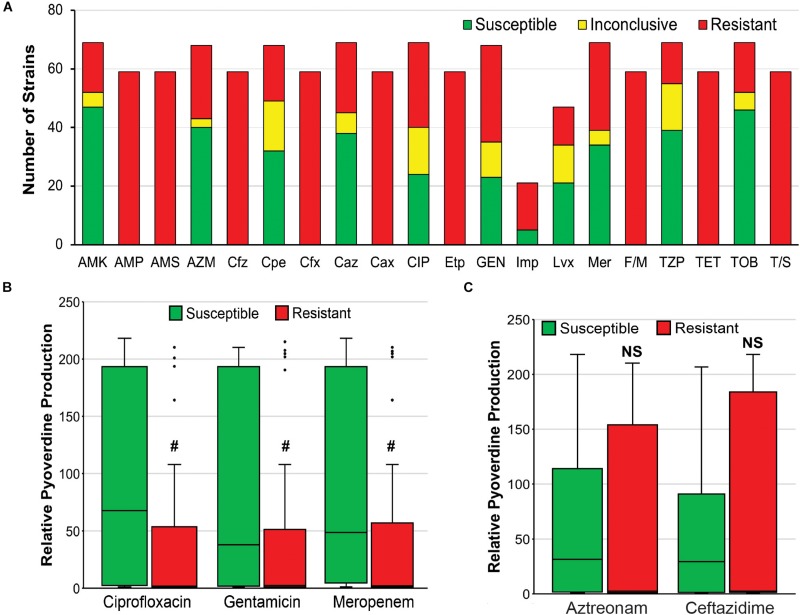
Antimicrobial resistance negatively correlates with pyoverdine production. **(A)** Susceptibility of 69 *P. aeruginosa* isolates to various antibiotics, including AMK, amikacin; AMP, ampicillin; AMS, ampicillin-sulbactam; AZM, aztreonam; Cfz, cefazolin; Cpe, cefepime; Cfx, cefoxitin; Caz, ceftazidime; Cax, ceftriaxone; CIP, ciprofloxacin; Etp, ertapenem; GEN, gentamicin; Imp, imipenem; Lvx, levofloxacin; Mer, meropenem; F/M, nitrofurantoin; TZP, piperacillin-tazobactam; TET, tetracycline; TOB, tobramycin; T/S, trimethoprim-sulfamethoxazole. **(B,C)** Histogram of pyoverdine production (normalized to that of PA14) in isolates that are susceptible or resistant to **(B)** ciprofloxacin, gentamicin, or meropenem, **(C)** aztreonam, or ceftazidime. *p*-values were calculated using Student’s *t*-test. ^#^*p* < 0.05.

Second, it is possible that there is a tradeoff between antimicrobial resistance and pyoverdine production, as has been observed for other energetically costly virulence factors in *P. aeruginosa* (Jain et al., 2004). We observed that resistance to three antimicrobials (ciprofloxacin, gentamicin, meropenem) correlated very well with reduced pyoverdine production (Figures 7A,B). However, this correlation was not observed with other antimicrobials (Figure 7C). More studies are needed to determine whether there may be a trade-off in the host between antimicrobial resistance and pyoverdine expression, and whether this phenomenon is specific to particular drugs.

Third, a number of metabolic changes are made to adapt to the CF lung, and these changes may result in the bacterium using alternative iron sources. For example, there is ample evidence that *P. aeruginosa* can and does use other forms of iron in the CF respiratory tract, including Fe^2+^ and heme, as progression of CF frequently causes hemoptysis, vastly increasing the concentration of iron-rich heme in the respiratory tract (Hare et al., 2012; Cornelis and Dingemans, 2013; Hunter et al., 2013; Konings et al., 2013; Marvig et al., 2014; Nguyen et al., 2014; Minandri et al., 2016).

A final possibility is that the sampling is merely insufficient to be representative. For example, one frequently cited study examined only two different time points in one individual and concluded that pyoverdine production was lost over time (Spencer et al., 2003). Although other, more comprehensive, studies have come to the same conclusion, it is worth remembering that the collection of *P. aeruginosa* strains infecting any particular patient with CF is likely to be heterogeneous and dynamic.

The link between pyoverdine and virulence has been clearly established in many contexts, including in *C. elegans* Liquid Killing and in murine infections. However, recent studies in a *Galleria mellonella* infection model demonstrated a more complicated relationship between pyoverdine and pathogenesis (Weigert et al., 2017). The addition of exogenous pyoverdine at low concentrations seemed to attenuate pathogen virulence. Higher concentrations, on the other hand, did seem to aggravate virulence. Kümmerli and colleagues recently performed a meta-analysis of studies that investigate *P. aeruginosa* virulence including pyoverdine-associated phenomena (Granato et al., 2016). This analysis showed that the magnitude of the pyoverdine impact varied considerably, depending on the infection context.

Even in *C. elegans*, where pyoverdine is generally the most important virulence determinant, the link between pathogenesis and pyoverdine can be complex. Preliminary next-generation sequencing data suggest that there are substantial differences even between highly virulent strains that produce pyoverdine. Key virulence factors, such as phenazine biosynthesis, type III secretion, and type IV pilus biogenesis vary. Within our survey of CF isolates, we observed isolates with considerable virulence but little to no pyoverdine, and *vice versa*. The factors that complicate the relationship between pyoverdine production and virulence remain under investigation in our lab. Their identification will be essential, since they may influence the efficacy of pyoverdine inhibitors.

The spread of antimicrobial resistance constitutes a clear and present threat, and the development of drugs that target virulence, whether for use as monotherapies or in addition to antimicrobials, is sorely needed. Antivirulents have been previously proposed for treating *P. aeruginosa*, although the targets have generally been chronic virulence factors, like quorum sensing and biofilm formation. Here we have shown that pyoverdine inhibitors (5-FC, 5-FUR, LK11) may be useful tools to combat MDR *P. aeruginosa* strains; these compounds effectively rescued infected mice (Figure 1A) and *C. elegans* (Figure 6) regardless of the pathogen’s antimicrobial resistance profile. Although the efficacy of these approaches against established infections requires further investigation, and it may ultimately be necessary to use a cocktail of antivirulents (e.g., biofilm inhibitors, quorum sensing disruptors, and pyoverdine blockers) to prevent the establishment of virulent infections, these molecules are a promising line of inquiry for a new method of mitigating infection.

## Data Availability

The datasets generated for this study are available on request to the corresponding author.

## Ethics Statement

The animal study was reviewed and approved by the Texas A&M University Institutional Animal Care and Use Committee (IACUC).

## Author Contributions

CC and NK conceived and designed the experiments. DK, AR, QC, and KS collected the data. DK, AR, QC, KS, CC, and NK analyzed the data. DK and NK wrote the manuscript. DK, AR, QC, CC, and NK edited the manuscript.

## Conflict of Interest Statement

The authors declare that the research was conducted in the absence of any commercial or financial relationships that could be construed as a potential conflict of interest.

## Footnotes

1 https://cellprofiler.org/
