# Supplementary material for: Pyoverdine-Dependent Virulence of Pseudomonas aeruginosa Isolates From Cystic Fibrosis Patients
Source: Front Microbiol. 2019 Sep 6;10:2048. doi: 10.3389/fmicb.2019.02048 (PMC6743535; doi:10.3389/fmicb.2019.02048)
Supplement: Supplementary file 1 [file Table_1.pdf]

| Strains | AMK | AMP | AMS | AZM | Cfz | Cpe | Cfx | Caz | Cax | CIP | Etp | GEN | Imp | Lvx | Mer | F/M | TZP | TET | TOB | T/S |
|---|---|---|---|---|---|---|---|---|---|---|---|---|---|---|---|---|---|---|---|---|
| PA2-15 | R | R | R | R | R | R | R | I | R | I | R | R |  | R | R | R | I | R | I | R |
| PA2-22 | R | R | R | R | R | R | R | R | R | R | R | R | R |  | R | R | R | R | S | R |
| PA2-23 | R | R | R | S | R | S | R | S | R | R | R | R | R |  | R | R | S | R | S | R |
| PA2-26 | S | R | R | S | R | S | R | S | R | I | R | I |  | I | R | R | S | R | S | R |
| PA2-29 | S | R | R | S | R | I | R | S | R | S | R | R |  | S | R | R | I | R | R | R |
| PA2-31 | S | R | R | S | R | S | R | S | R | S | R | R |  | S | I | R | S | R | R | R |
| PA2-34 | S | R | R | R | R | I | R | R | R | S | R | S |  | S | S | R | R | R | S | R |
| PA2-35 | S |  |  | S |  | S |  | S |  | R |  | S | S |  | S |  | S |  | S |  |
| PA2-38 | S | R | R | S | R | S | R | S | R | I | R | I |  | I | I | R | S | R | S | R |
| PA2-39 | S | R | R | R | R | I | R | I | R | R | R | S |  | I | R | R | R | R | S | R |
| PA2-44 | R | R | R | S | R | S | R | S | R | R | R | R | R |  | S | R | S | R | R | R |
| PA2-45 | S | R | R | I | R | S | R | R | R | I | R | R |  | I | R | R | I | R | R | R |
| PA2-46 | I | R | R | S | R | S | R | S | R | R | R | R |  | R | S | R | S | R | S | R |
| PA2-47 | S | R | R | S | R | I | R | S | R | I | R | I |  | I | R | R | S | R | S | R |
| PA2-51 | I | R | R | S | R | I | R | I | R | S | R | R |  | S | S | R | S | R | I | R |
| PA2-53 | S | R | R | S | R | R | R | S | R | R | R | S |  | I | S | R | S | R | S | R |
| PA2-58 | S | R | R | S | R | S | R | R | R | S | R | R |  | S | S | R | S | R | I | R |
| PA2-59 | S | R | R | R | R | R | R | I | R | I | R | R |  | I | R | R | I | R | I | R |
| PA2-61 | S | R | R | I | R | S | R | R | R | S | R | R |  | S | S | R | S | R | I | R |
| PA2-63 | R | R | R | S | R | R | R | S | R | R | R | R | R |  | I | R | S | R | R | R |
| PA2-66 | I | R | R | S | R | S | R | S | R | S | R | I |  | S | S | R | S | R | S | R |
| PA2-67 | S | R | R | S | R | S | R | S | R | S | R | S |  | S | S | R | S | R | S | R |
| PA2-72 | S | R | R | S | R | S | R | S | R | S | R | S |  | S | S | R | S | R | S | R |
| PA2-78 | S | R | R | S | R | S | R | S | R | S | R | R |  | S | S | R | S | R | R | R |
| PA2-80 | S | R | R | S | R | I | R | S | R | R | R | S | R |  | R | R | S | R | S | R |
| PA2-87 | S | R | R | R | R | I | R | S | R | R | R | S |  | R | S | R | I | R | S | R |
| PA2-88 | S | R | R | S | R | S | R | S | R | I | R | R |  | I | S | R | S | R | S | R |
| PA2-89 | R |  |  | S |  | R |  | R |  | R |  | I | R |  | R |  | I |  | S |  |
| PA2-9 | S | R | R | S | R | S | R | S | R | S | R | S |  | S | S | R | S | R | S | R |
| PA2-94 | S |  |  | R |  | R |  | R |  | S |  | R | R |  | R |  | R |  | S |  |
| PA2-95A | S | R | R | S | R | S | R | S | R | S | R | R |  | S | S | R | S | R | R | R |
| PA2-99 | S | R | R | S | R | S | R | S | R | R | R | I |  | R | R | R | S | R | R | R |
| PA3-13 | R | R | R | R | R | R | R | S | R | R | R | R | R |  | R | R | S | R | R | R |
| PA3-15 | R |  |  | S |  | S |  | S |  | S |  | R | S |  | S |  | S |  | R |  |
| PA3-16 | S |  |  | S |  | S |  | S |  | R |  | I | S |  | S |  | S |  | S |  |
| PA3-17 | R | R | R | I | R | R | R | R | R | I | R | R | R |  | R | R | I | R | R | R |
| PA3-18 | S | R | R | R | R |  | R | R | R | I | R | R |  | S | S | R | R | R | R | R |
| PA3-22 | S | R | R | S | R | S | R | S | R | I | R | R |  | I | S | R | S | R | I | R |

| | | | | | | | | | | | | | | | | | | | | |
|---|---|---|---|---|---|---|---|---|---|---|---|---|---|---|---|---|---|---|---|---|
| PA3-23 | R | R | R | R | R | R | R | R | R | R | R | R | R | | R | R | I | R | S | R |
| PA3-25 | S | R | R | R | R | R | R | R | R | S | R | S | | S | R | R | R | R | S | R |
| PA3-27 | R | R | R | R | R | I | R | S | R | R | R | R | | R | I | R | I | R | R | R |
| PA3-28 | R | | | S | | S | | S | | I | | R | R | | R | | S | | S | |
| PA3-29 | S | R | R | R | R | I | R | R | R | S | R | S | | S | S | R | I | R | S | R |
| PA3-31 | S | R | R | R | R | I | R | S | R | I | R | S | | I | S | R | I | R | S | R |
| PA3-34B | R | R | R | S | R | I | R | S | R | R | R | R | R | | R | R | S | R | R | R |
| PA3-37 | R | | | R | | R | | R | | S | | R | R | | R | | R | | S | |
| PA3-39 | S | R | R | S | R | S | R | R | R | S | R | I | | R | R | R | R | R | S | R |
| PA3-40 | S | R | R | R | R | R | R | R | R | R | R | S | | R | R | R | R | R | S | R |
| PA3-45 | S | R | R | S | R | S | R | S | R | S | R | S | | S | S | R | S | R | S | R |
| PA3-46 | R | | | R | | R | | R | | R | | R | S | | S | | I | | R | |
| PA3-47 | R | | | R | | R | | R | | I | | R | R | | R | | R | | S | |
| PA3-48 | S | R | R | R | R | R | R | R | R | R | R | S | | R | R | R | R | R | S | R |
| PA3-49 | S | R | R | R | R | I | R | I | R | R | R | | | R | R | R | I | R | S | R |
| PA3-5 | I | | | S | | I | | R | | I | | S | R | | I | | S | | S | |
| PA3-51 | S | R | R | S | R | S | R | S | R | R | R | R | | | S | R | S | R | S | R |
| PA3-52 | S | R | R | S | R | I | R | S | R | R | R | S | S | | S | R | S | R | S | R |
| PA3-54 | S | R | R | S | R | S | R | R | R | S | R | I | | S | S | R | S | R | R | R |
| PA3-57 | S | R | R | S | R | S | R | R | R | S | R | R | | S | S | R | S | R | R | R |
| PA3-6 | R | R | R | S | R | S | R | S | R | R | R | R | R | | R | R | S | R | S | R |
| PA3-61 | S | R | R | R | R | R | R | I | R | R | R | S | | R | R | R | I | R | S | R |
| PA3-66 | I | R | R | | R | I | R | I | R | R | R | R | | R | R | R | I | R | S | R |
| PA3-9 | S | R | R | R | R | R | R | R | R | S | R | S | | S | R | R | R | R | S | R |
| PA5-31 | S | R | R | R | R | I | R | S | R | I | R | S | | I | S | R | I | R | S | R |
| PA5-40 | S | R | R | R | R | R | R | R | R | R | R | I | | R | R | R | R | R | S | R |
| PA5-45 | S | R | R | S | R | S | R | S | R | S | R | S | | S | S | R | S | R | S | R |
| PA5-51 | S | R | R | S | R | S | R | S | R | I | R | I | | I | S | R | S | R | S | R |
| PA5-52 | S | R | R | S | R | S | R | S | R | R | R | S | | R | S | R | S | R | S | R |
| PA5-54 | S | R | R | S | R | S | R | S | R | R | R | I | | I | S | R | S | R | S | R |
| PALF-05 | S | R | R | R | R | I | R | R | R | S | R | S | | S | S | R | R | R | S | R |

**Table S1. Table 1. Antimicrobial susceptibility of commonly used antibiotics against CF *P. aeruginosa* isolates.** AMK: Amikacin; AMP: Ampicillin; AMS: Ampicillin-Sulbactam; AZM: Aztreonam; Cfz: Cefazolin; Cpe: Cefepime; Cfx: Cefoxitin; Caz: Ceftazidime; Cax: Ceftriaxone; CIP: Ciprofloxacin; Etp: Ertapenem; GEN: Gentamicin; Imp: Imipenem; Lvx: Levofloxacin; Mer: Meropenem; F/M: Nitrofurantoin; TZP: Piperacillin-Tazobactam; TET: Tetracycline; TOB: Tobramycin; T/S: Trimethoprim-Sulfamethoxazole. S, I, and R indicate sensitivity, intermediate resistance, and complete resistance to the tested antimicrobial, respectively.
