# Supplementary material for: Pyoverdine-Dependent Virulence of Pseudomonas aeruginosa Isolates From Cystic Fibrosis Patients
Source: Front Microbiol. 2019 Sep 6;10:2048. doi: 10.3389/fmicb.2019.02048 (PMC6743535; doi:10.3389/fmicb.2019.02048)
Supplement: Supplementary file 2 [file Image_1.pdf]

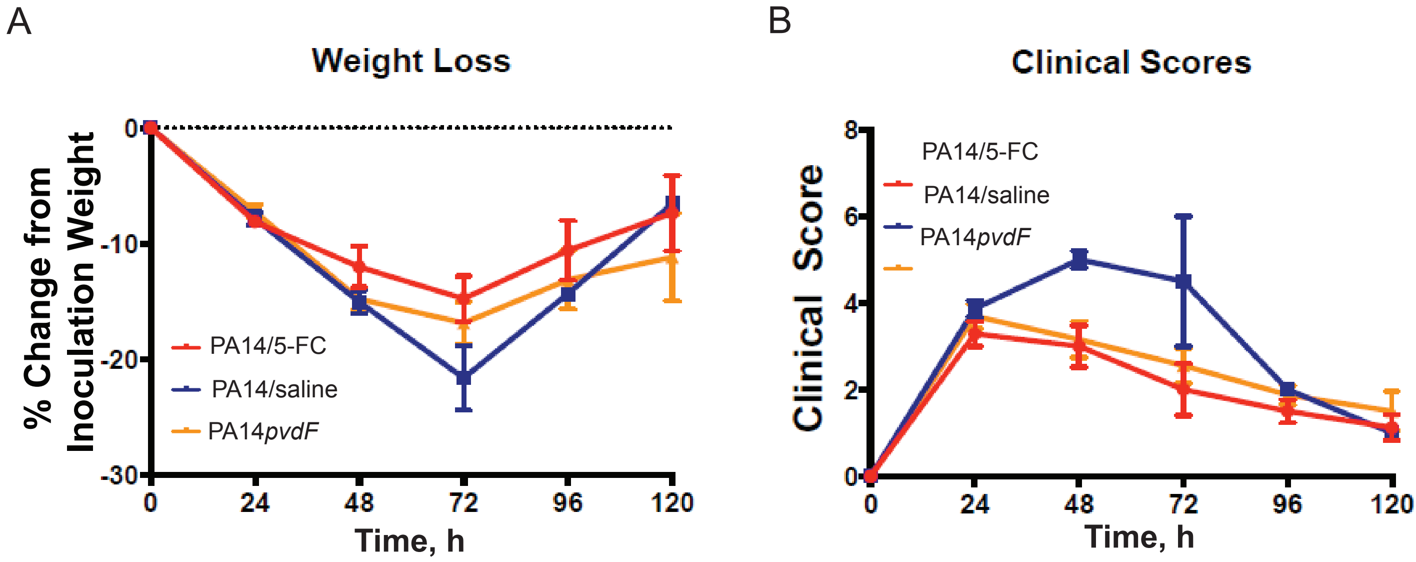

Figure S1. Morbidity of mice exposed to wild-type *P. aeruginosa* with or without the pyoverdine inhibitor 5-fluorocytosine or pyoverdine biosynthetic mutant. (A) Weights of *P. aeruginosa*-infected mice over time. Mice were treated with WT PA14/saline, PA14*pvdF*/saline, or WT PA14/5-FC. **(B)** Clinical scores of *P. aeruginosa*-infected mice over time. Higher clinical scores indicated lack of movement, ruffled fur, and/or hunched posture.
