# Supplementary material for: Pyoverdine-Dependent Virulence of Pseudomonas aeruginosa Isolates From Cystic Fibrosis Patients
Source: Front Microbiol. 2019 Sep 6;10:2048. doi: 10.3389/fmicb.2019.02048 (PMC6743535; doi:10.3389/fmicb.2019.02048)
Supplement: Supplementary file 3 [file Image_2.pdf]

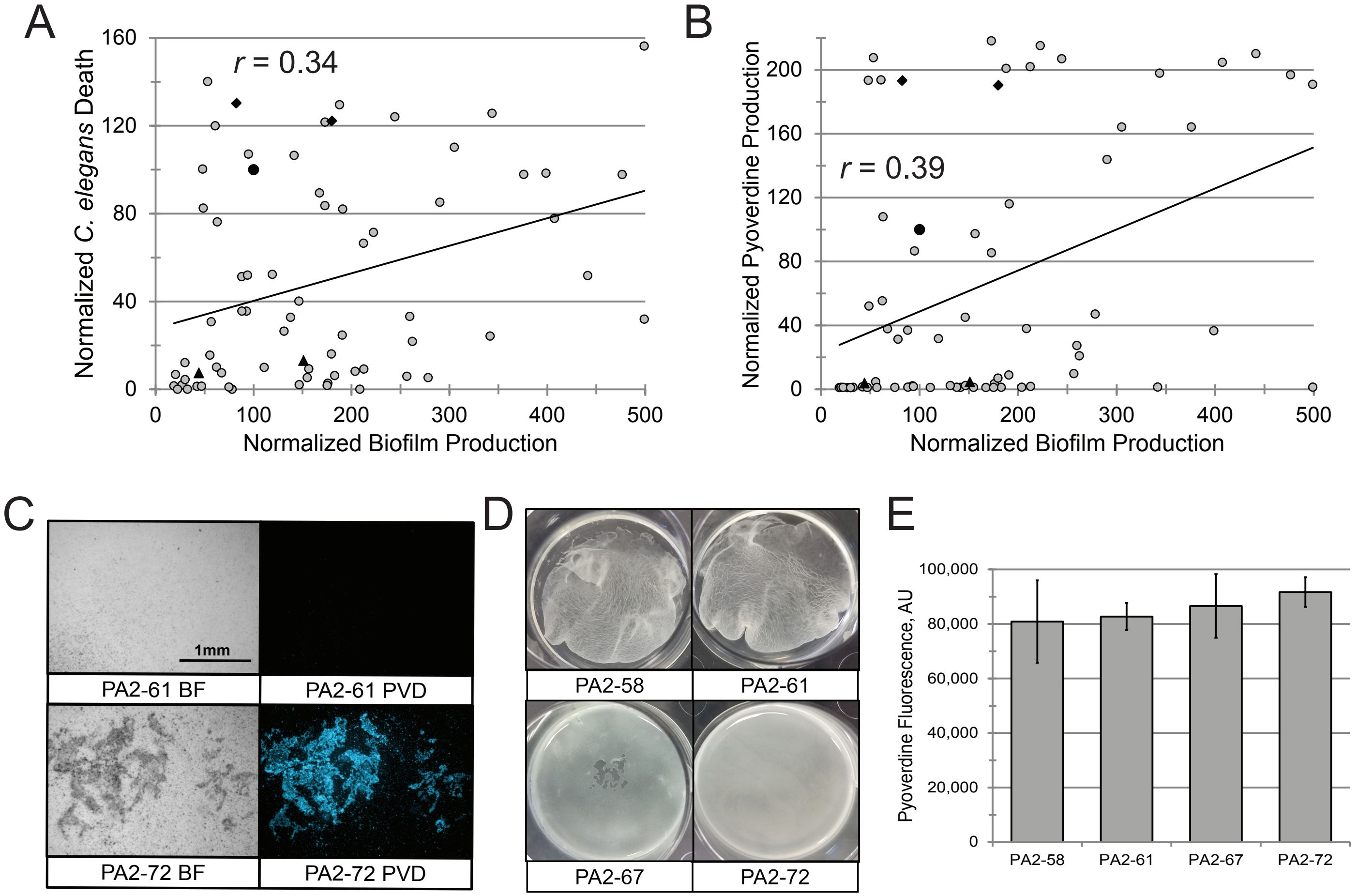

Figure S2. Cell aggregation in *P. aeruginosa* isolates drives pyoverdine production. (A, B) Correlation between biofilm production and *C. elegans* death during Liquid Killing **(A)** or between the production of biofilm and pyoverdine **(B).** All data were normalized to PA14. Black diamonds and triangles represent the high- and low-virulence isolates selected for further study. Each point represents the average of at least two biological replicates. **(C)** Fluorescent micrographs of cells attached to the culture vessel for two isolates that form biofilms (PA2-67, PA2-72) and two isolates that form unconventional cell aggregates (PA2-58, PA2-61). Cells were visualized using a pyoverdine-specific fluorescence filter. **(D)** Image of *P. aeruginosa* isolates grown statically for 16 h in 6-well plates. **(E)** Pyoverdine fluorescence in the bacterial cultures. Error bars represent SEM between two biological replicates.
