# Supplementary material for: Pyoverdine-Dependent Virulence of Pseudomonas aeruginosa Isolates From Cystic Fibrosis Patients
Source: Front Microbiol. 2019 Sep 6;10:2048. doi: 10.3389/fmicb.2019.02048 (PMC6743535; doi:10.3389/fmicb.2019.02048)
Supplement: Supplementary file 4 [file Image_3.pdf]

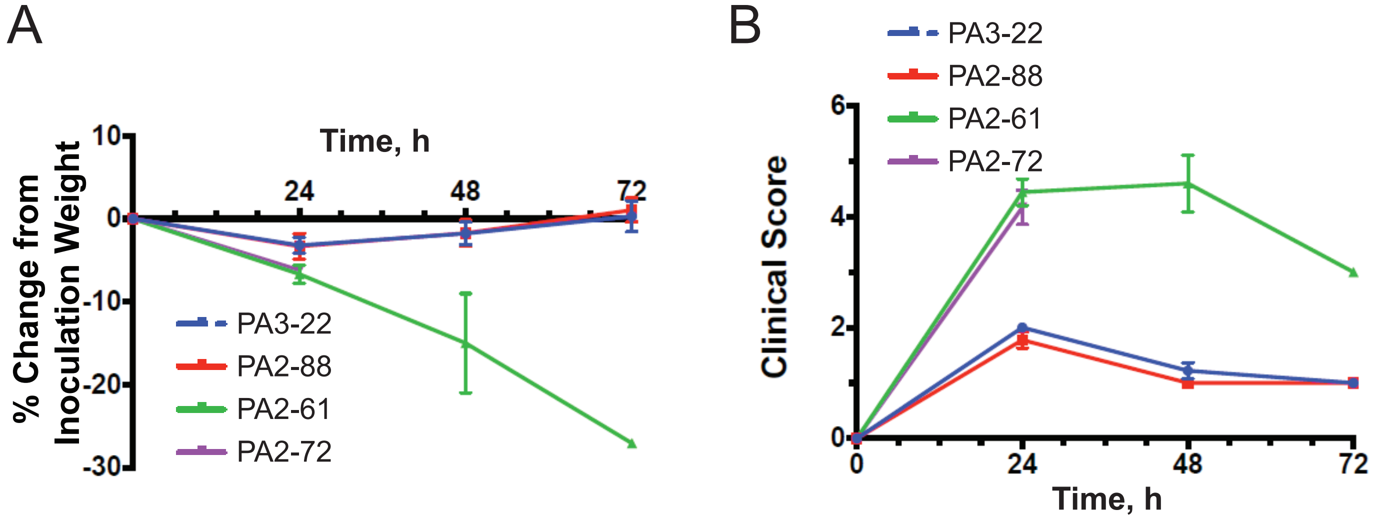


**Figure S3. Morbidity of mice exposed to highly virulent and avirulent *P. aeruginosa* isolates. (A)** Weights of *P. aeruginosa*-infected mice over time. Mice were infected with two highly virulent (PA2-61, PA2-72) and two avirulent (PA2-88, PA3-22) isolates of *P. aeruginosa*. **(B)** Clinical scores of *P. aeruginosa*-infected mice over time. Higher clinical scores indicated lack of movement, ruffled fur, and/or hunched posture.
