# Supplementary material for: Pyoverdine-Dependent Virulence of Pseudomonas aeruginosa Isolates From Cystic Fibrosis Patients
Source: Front Microbiol. 2019 Sep 6;10:2048. doi: 10.3389/fmicb.2019.02048 (PMC6743535; doi:10.3389/fmicb.2019.02048)
Supplement: Supplementary file 5 [file Image_4.pdf]

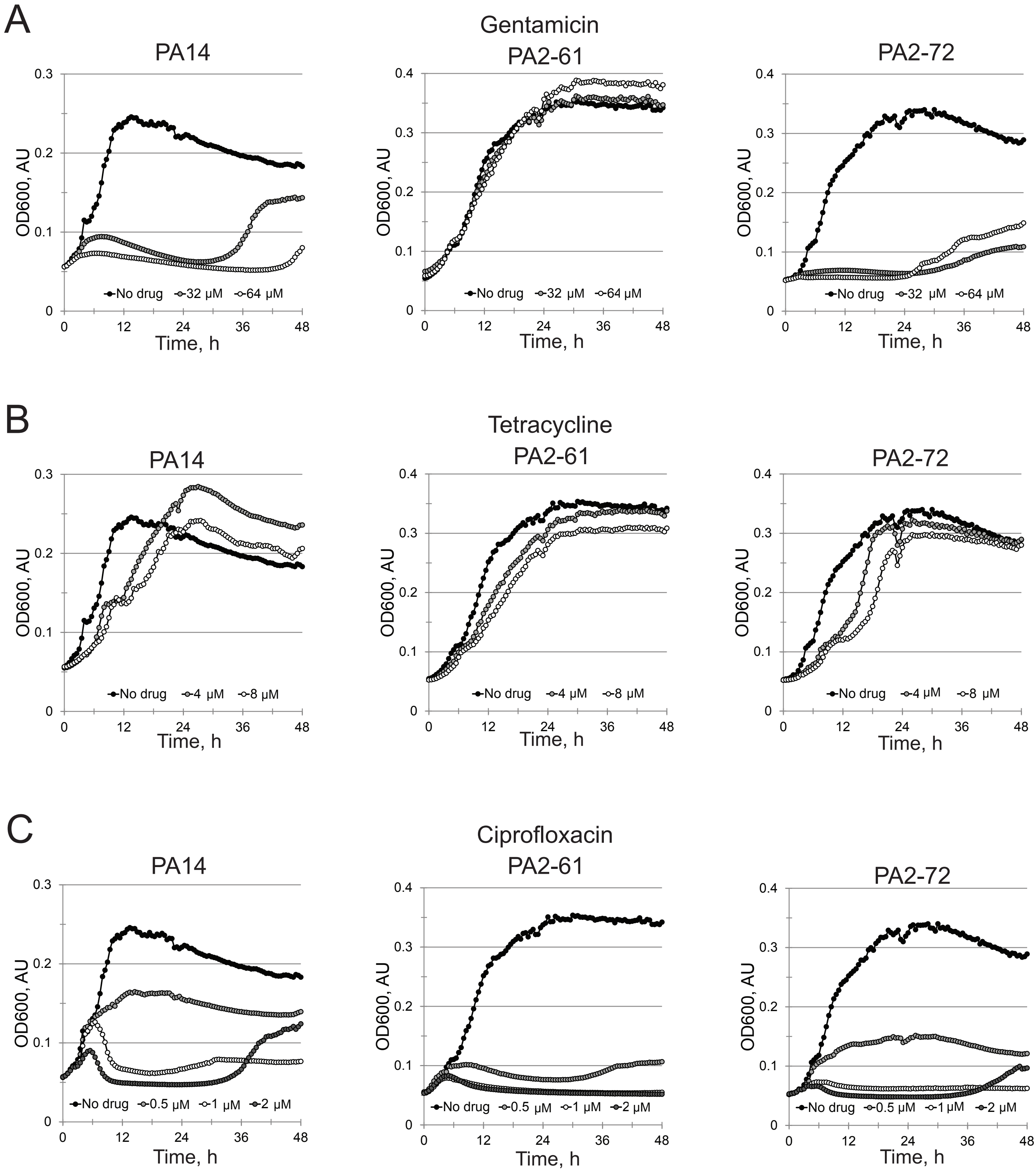

Figure S4. Effect of antimicrobials on the growth of highly virulent *P. aeruginosa* isolates. Growth curves for *P. aeruginosa* PA14, PA2-61, or PA2-72 in the presence of varying concentrations of **(A)** gentamicin, **(B)** tetracycline, or **(C)** ciprofloxacin. OD_{600} readings were taken every 30 min for 48 h.
