# Supplementary material for: Pyoverdine-Dependent Virulence of Pseudomonas aeruginosa Isolates From Cystic Fibrosis Patients
Source: Front Microbiol. 2019 Sep 6;10:2048. doi: 10.3389/fmicb.2019.02048 (PMC6743535; doi:10.3389/fmicb.2019.02048)
Supplement: Supplementary file 6 [file Image_5.pdf]

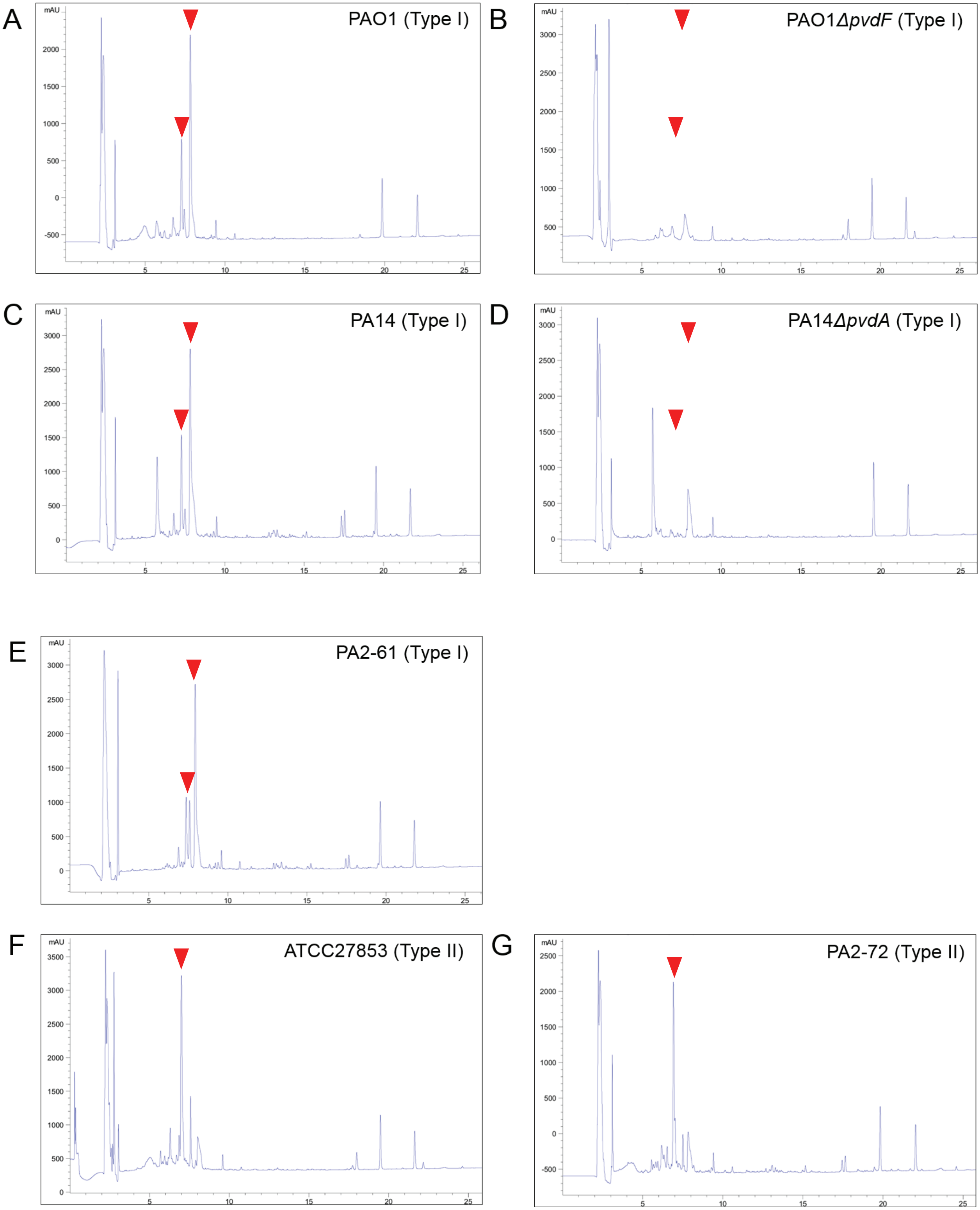
A
PAO1 (Type I)
B
PAO1ΔpvdF (Type I)
C
PA14 (Type I)
D
PA14ΔpvdA (Type I)
E
PA2-61 (Type I)
F
ATCC27853 (Type II)
G
PA2-72 (Type II)
mAU

H

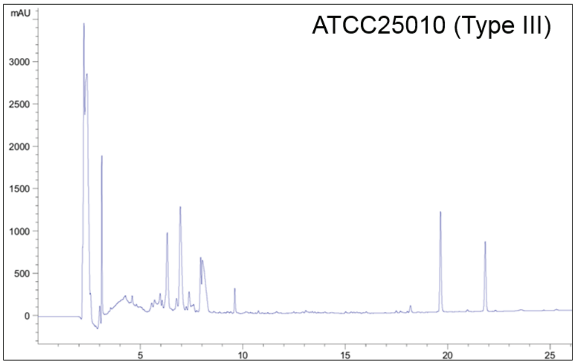

I

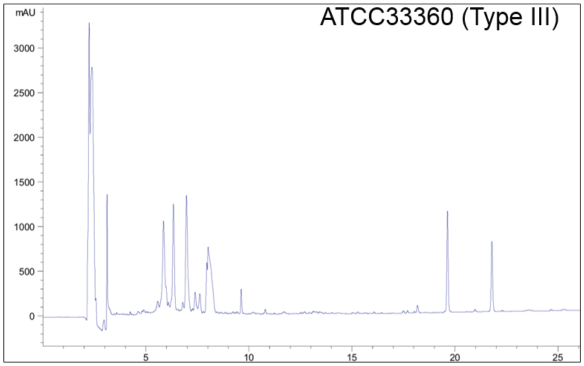

Figure S5. *P. aeruginosa* isolates produce different types of pyoverdines. HPLC spectra of pyoverdine-rich *P. aeruginosa* filtrates for strains with defined siderotypes and uncharacterized isolates. Pyoverdine type I: **(A, B)** PAO1, **(C, D)** PA14, **(E)** PA2-61. Pyoverdine type II: **(F)** ATCC27853, **(G)** PA2-72. Pyoverdine type III: **(H)** ATCC25010, **(I)** ATCC33360. Red arrows point to putative pyoverdine peaks.
